# Getting rid of ‘rain’ and ‘stars’: Mitigating inhibition effects on ddPCR data analysis, the case study of the invasive crayfish *Pacifastacus leniusculus* in the streams of Luxembourg

**DOI:** 10.1371/journal.pone.0275363

**Published:** 2022-11-16

**Authors:** David Porco, Sylvie Hermant, Chanistya Ayu Purnomo, Mario Horn, Guy Marson, Guy Colling

**Affiliations:** 1 Musée National d’histoire Naturelle, Life Science Department—Invertebrate Zoology, Population Biology and Evolution, Luxembourg, Luxembourg; 2 Fondation Faune Flore, Luxembourg, Luxembourg; Texas A&M Health Science Center, UNITED STATES

## Abstract

ddPCR is becoming one of the most widely used tool in the field of eDNA-based aquatic monitoring. Although emulsion PCR used in ddPCR confers a partial mitigation to inhibition due to the high number of reactions for a single sample (between 10K and 20K), it is not impervious to it. Our results showed that inhibition impacts the amplitude of fluorescence in positive droplets with a different intensity among rivers. This signal fluctuation could jeopardize the use of a shared threshold among samples from different origin, and thus the accurate assignment of the positive droplets which is particularly important for low concentration samples such as eDNA ones: amplification events are scarce, thus their objective discrimination as positive is crucial. Another issue, related to target low concentration, is the artifactual generation of high fluorescence droplets (‘stars’). Indeed, these could be counted as positive with a single threshold solution, which in turn could produce false positive and incorrect target concentration assessments. Approximating the positive and negative droplets distribution as normal, we proposed here a double threshold method accounting for both high fluorescence droplets (‘stars’) and PCR inhibition impact in delineating positive droplets clouds. In the context of low concentration template recovered from environmental samples, the application of this method of double threshold establishment could allow for a consistent sorting of the positive and negative droplets throughout ddPCR data generated from samples with varying levels of inhibitor contents. Due to low concentrations template and inhibition effects, Quantasoft software produced an important number of false negatives and positive comparatively to the double threshold method developed here. This case study allowed the detection of the invasive crayfish *P*. *leniusculus* in 32 out of 34 sampled sites from two main rivers (Alzette and Sûre) and five of their tributaries (Eisch, Attert, Mamer, Wiltz and Clerve).

## Introduction

Digital PCR (dPCR) is a molecular technique that can achieve high sensitivity detection and absolute quantification of template concentration in samples [1, 2]. Digital droplet PCR (ddPCR) is one of the iterations of this technique. It relies on a water/oil emulsion partitioning of PCR mix in thousands of independent reactions that are counted as positive or negative events. The proportion of positive events enables the application of Poisson binomial distribution statistics leading to the assessment of the target concentration in the sample [3]. Originally, and to date, the technique was used in the field of medical diagnostics (e.g. [4, 5]) and in transgenic DNA quantification in GMO (e.g. [6, 7]). However, it has now found a broad application in eDNA-based detection of organisms in various aquatic environments [8–15] and was also specifically targeted at crayfish detection, either native or invasive species [16–18].

Although ddPCR is inhibition resistant due to the high number of amplification events (optimally around 20 000), is not insensitive to it [19, 20]. The inhibition factor is a combination of local and often time-dependent occurrence of chemical compounds derived from sediments and the degradation of animal and plant organic matter, such as humic, fulvic and tannic acids [21, 22]. Inhibition has been repeatedly pointed out as a critical factor hindering amplification and thus preventing consistent detections in eDNA studies because of false negatives or uncertainties in DNA quantification (e.g. [21–25]). In ddPCR, inhibition decreases the fluorescence of positive droplets [19, 20] which could then be falsely identified as ‘rain’ i.e. droplets with intermediate fluorescence values between positive and negative ones.

Various techniques and software were used in order to decide about the threshold to be applied to ddPCR results (e.g. the Bio-Rad software Quantasoft, Definite the rain [26], Umbrella [27], ddPCRquant [28]). However, they can yield poor to suboptimal results when applied directly or through the use of positive control calibration to eDNA samples due to low concentration templates and potential inhibitor amounts [29].

Here, while proposing an eDNA-based survey of the invasive crayfish *Pacifastacus leniusculus* in the streams of Luxembourg, we introduced a simple methodology consistently accounting for the specific inhibition factor in several surveyed rivers. The goal was to achieve higher accuracy in detection and DNA target concentration assessment.

## Material and method

### Crayfish material

*Pacifastacus leniusculus* specimens were fished in the river Alzette (Hesperange Park, lat: 49.57070, long: 6.15697). The water agency (AGE) from Luxembourg delivered a fishing permit for this sampling (Permit n°91125 CD/ne). Genomic DNA was extracted from leg’s muscle tissue from these specimens with a Qiagen DNeasy Blood & Tissue Kit following the manufacturer’s instructions.

### Water sampling

**Fig 1:**
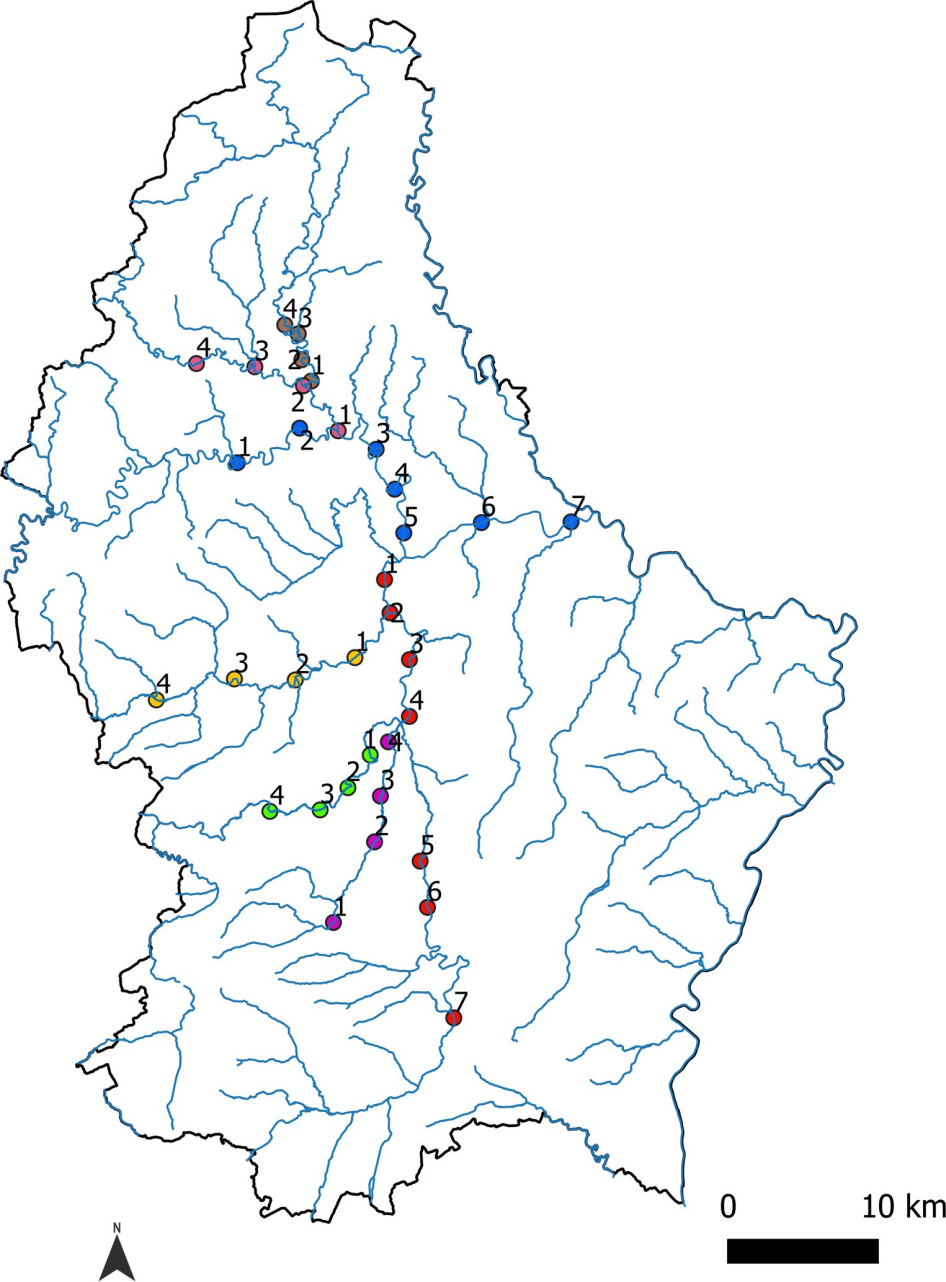
Localization of the 34 sampling sites. The colors of the dots represent the different rivers sampled (Red = Alzette, Blue = Sure, Green = Eisch, Brown = Clerve, Pink = Wiltz, Orange = Attert, Purple = Mamer).

Two of the main rivers in Luxembourg along with some of their tributaries were sampled: Alzette (Eisch, Attert, Mamer) and Sûre (Wiltz, Clerve). In total, a network of 34 sampling sites was analysed, comprising seven sites per main river (Alzette and Sûre) and four for each of their tributaries (Eisch, Attert, Mamer, Wiltz, Clerve) (Fig 1). At each site, ten replicates of 500 ml water were collected with screw cap PET bottles. Five replicates were sampled at 30 cm and five at 1.50 m from the bank. On each line (30 cm and 1.50 m), replicates were 50 cm apart from each other. Sampling depth ranged from 5 and 10 cm from the surface. Sampling took place during the mating season i.e. the first half of October, which is also a time when heavy rains have not yet overcharged the targeted rivers. These conditions were chosen in order to maximize the DNA concentration levels and thus detection probability.

Each water sample (stored at 4°C for a maximum of 3 h before processing) was filtered with a peristaltic pump (Masterflex L/S Standard Pump Head) connected to a column driller (500 W) through a 0.45 μm nitrocellulose membrane (Nalgene analytical funnel). The resulting filter was immediately stored in 800 μl ATL Qiagen lysis buffer at 4°C in 1.5 ml microfuge tubes and then frozen at -20°C back in the lab. For Alzette, Attert, Eisch and Mamer rivers, the filtration could not be performed for the entire 500 ml volume in some of the replicates because of the presence of high amounts of suspended sediments in the water column (S1 Table). For each site, a negative control was established in order to detect any possible cross-contamination: it consisted in a 500 ml bottle of distilled water brought into the field and that underwent the same storage and filtration process as the samples.

### DNA extraction

DNA was extracted from filters using a Qiagen DNeasy Blood & Tissue Kit with a volume-adapted protocol: following a thorough shredding of the filter directly in the tube with clean scissors, 80 μl ProK were added to the lysis buffer used for filter preservation. After an overnight incubation at 56°C, 600 μl of the lysis solution was recovered and mixed with 600 μl AL Qiagen lysis buffer (10 minutes incubation at 56°C) and 600 μl ethanol. Subsequently to a homogenization by mixing, the solution was transferred to Qiagen DNeasy 96 plates. The rest of the protocol followed the manufacturer’s instructions. DNA extracts were eluted in 100 μl of AE Qiagen elution buffer heated at 56°C. In order to monitor any potential sample cross contamination, negative controls (ATL buffer with proteinase K) were extracted along with each series of samples.

### ddPCR

The extracts were processed for ddPCR and read on a Bio–Rad QX200 suite according to the manufacturer’s instructions (https://www.bio-rad.com/webroot/web/pdf/lsr/literature/Bulletin_6407.pdf). The evagreen mix was used along with the specific primer set previously designed for *P*. *leniusculus* [30]. The PCR cycling program followed the manufacturer’s instructions with an annealing temperature of 60°C and 50 cycles. The reaction mix was composed of 11 μl Evagreen Bio–Rad Supermix and 200 nM primer, completed with water to 21 μl and added to 1 μl template DNA or water as a negative control. Products from duplicates were recovered from emulsion according to Bio-Rad’s instructions (https://www.bio-rad.com/webroot/web/pdf/lsr/literature/Bulletin_6407.pdf), rePCRed using the same primer set at 0.25 μM concentration with 2 μl of 1/10 diluted recovered PCR products added to 15 μl of GoTaq mastermix (Promega) and completed with water to 25 μl. Temperature program was 95°C for 3 min followed by 35 cycles of denaturation at 95°C for 30 s, annealing at 60°C for 30 s, extension at 72°C for 60 s and a final extension at 72°C for 5 min. The amplicons were purified with Agencourt AMPure XP and sequenced both directions with Big Dye terminator. A second purification with Agencourt CleanSEQ eliminated the excess dye-terminators. The sequencing was run on a Sanger sequencer ABI 3730 XL. The sequences obtained were aligned to 168 *P*. *leniusculus* COI haplotypes available in Genbank (accessions: EU921148.1, JF437995.1—JF437998.1, KU603480.1, KU603492.1—KU603496.1, KU603526.1, KU603527.1, KU603534.1, KU603535.1, KY947312.1—KY947338.1, ON058995—ON059120).

### Inhibition impact experiment

To measure the level of inhibition impacting the ddPCR assay in the different rivers, positive controls (1 μl of *P*. *leniusculus* genomic DNA at 15 ng/μl) were spiked with eDNA samples from each river. The ddPCR reactions for each eDNA river extracts were prepared with two different amounts of eDNA extract: 0,5 μl and 1 μl per reaction replacing an equivalent amount of water in the PCR mix. For each treatment (i.e. 0.5 and 1 μl eDNA extract), six replicates where used for the main rivers (Alzette and Sûre) and three for each of the tributaries (Eisch, Attert, Mamer, Wiltz, Clerve). Twenty-three replicates were used for genomic DNA positive controls with no eDNA extracts.

### Data treatment as a countermeasure

**Fig 2:**
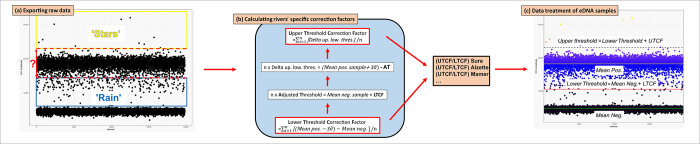
Schematic workflow of the data treatment. (a) Export of the raw fluorescence data from positive control (genomic extract at 15 ng/μl) spiked with two different amount of river eDNA extract in order to define the part of the signal that can be accounted as ‘rain’ and stars’; (b) from these data, for each rivers (n = 6 for the main rivers Alzette and Sûre and n = 3 for tributaries Eisch, Attert, Mamer, Wiltz, Clerve), the descriptive statistics (Mean pos. = Mean fluorescence value for the positive droplets cloud, ∂ = standard deviation for the positive cloud and mean neg. = Mean fluorescence value for the negative droplets cloud) were calculated and, in turn, used to estimate the two threshold correction factors (Upper Threshold Correction Factor (UTCF) and Lower Threshold Correction Factor (LTCF); (c) River specific threshold correction factors were applied to the analysis of the samples in order to consistently detect and measure target DNA concentrations.

Raw fluorescence amplitude data obtained from the Inhibition impact experiment with 1 μl eDNA extract were exported with Quantasoft 1.7.4 (Bio-Rad). From these, approximating the distribution of the droplets in each cloud as normal [31], two corrections factors were calculated for each river: an Upper and a Lower Threshold Correction Factor (respectively UTCF and LTCF–Fig 2, S2 Table). These correction factors were then used to define consistently and specifically an upper and a lower threshold in order to delineate the cloud of positive droplets in the samples processed from each river: LCTF was summed with the mean fluorescence of the negative cloud to calculate the lower threshold, and then the lower threshold was added with UTCF to obtain the upper threshold (Fig 2, S2 Table). Thus, for each sample, the calculation of these two thresholds with LTCF and UTCF is based on the mean fluorescence of the negative droplets cloud which is always the strongest signal in eDNA data. Using these defined thresholds adapted to each sample baseline fluorescence shifting and the inhibition level in the river, absolute concentrations of the samples were recovered.

### Limit of Blank (LoB), Limit of Detection (LoD) and LoQ (Limit of Quantification)

After applying the double threshold steps described in the previous paragraph, a detection and a quantification filtering was used on the obtained measurements. Several currently used metrics were calculated [32]:

1. the Limit of Blank (LoB), which is the highest concentration that can be found in no template controls (NTC), was determined from 78 NTC.
$$LoB=mean_{NTC}+3\;SD_{NTC}$$
2. the Limit of Detection (LoD), which is the lowest concentration of target DNA that can be detected with sufficient confidence, was derived from LoB using the standard deviation from the lowest detectable concentration of positive control.
$$LoD=LoB+3\;(SD_{low\;concentration\;sample})$$
  The confidence interval chosen for LoB and LoD calculations was 99.73%, this in order to strongly minimize false positives.

The LoQ (Limit of Quantification) i.e. the lowest concentration that can be assessed in 90% replicates, was established from a dilution series of genomic DNA (ranging from 15 ng/μl to 15x10-8 ng/μl) with 5 replicates for each concentration [33].

### Comparison with Quantasoft results

To monitor the comparative efficiency of the double threshold method proposed here, the whole dataset was also analysed with the Bio-Rad software Quantasoft. The results were compared for the global number of false negative and positive it could generate. For this, the double threshold results were used as a reference as they took into account the significant effects of inhibition measured in the sampled rivers. The global distributions of the DNA concentrations obtained with the two methods were compared with an asymptotic two-sample Kolmogorov-Smirnov test. In addition, the DNA concentrations obtained from the two methods for the sites’ replicates in each river were tested for linear correlation. This to assess the equivalence of the results produced from the two methods in terms of relative concentration pattern. It was paralleled with the relative inhibition effect found in each river based on the mean fluorescence amplitude between the lower and upper threshold calculated from the inhibition experiment. The reference for the relative inhibition effect was the mean amplitude between these thresholds found in the positive controls.

### Statistics and data analysis

Statistical analyses and correction factors calculation were conducted in the RStudio V1.2.5042 environment [34] and graphs plotted with the packages ggpubr and ggplot2 [35]. Maps were made with QGIS 3.2.1 [36].

## Results

### Inhibition experiments

**Fig 3:**
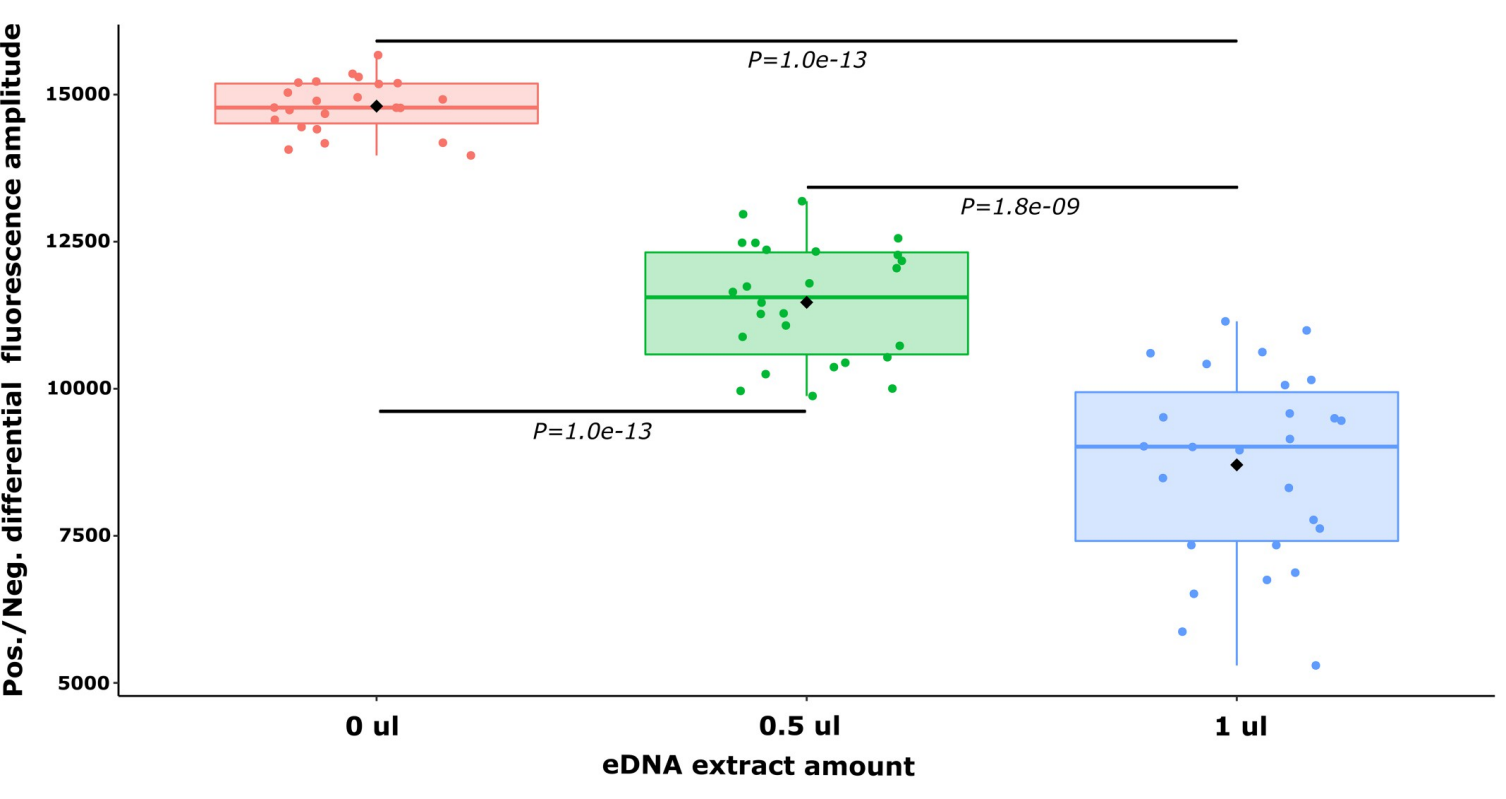
Boxplot representation of the differential fluorescence amplitudes between the mean fluorescence of the positive and the negative droplet clouds for different amounts of eDNA samples (0, 0.5 and 1 μl) spiked into positive controls (*P*. *leniusculus* genomic DNA). Pairwise Wilcoxon test P values with Holm correction are indicated on horizontal bars.

**Fig 4:**
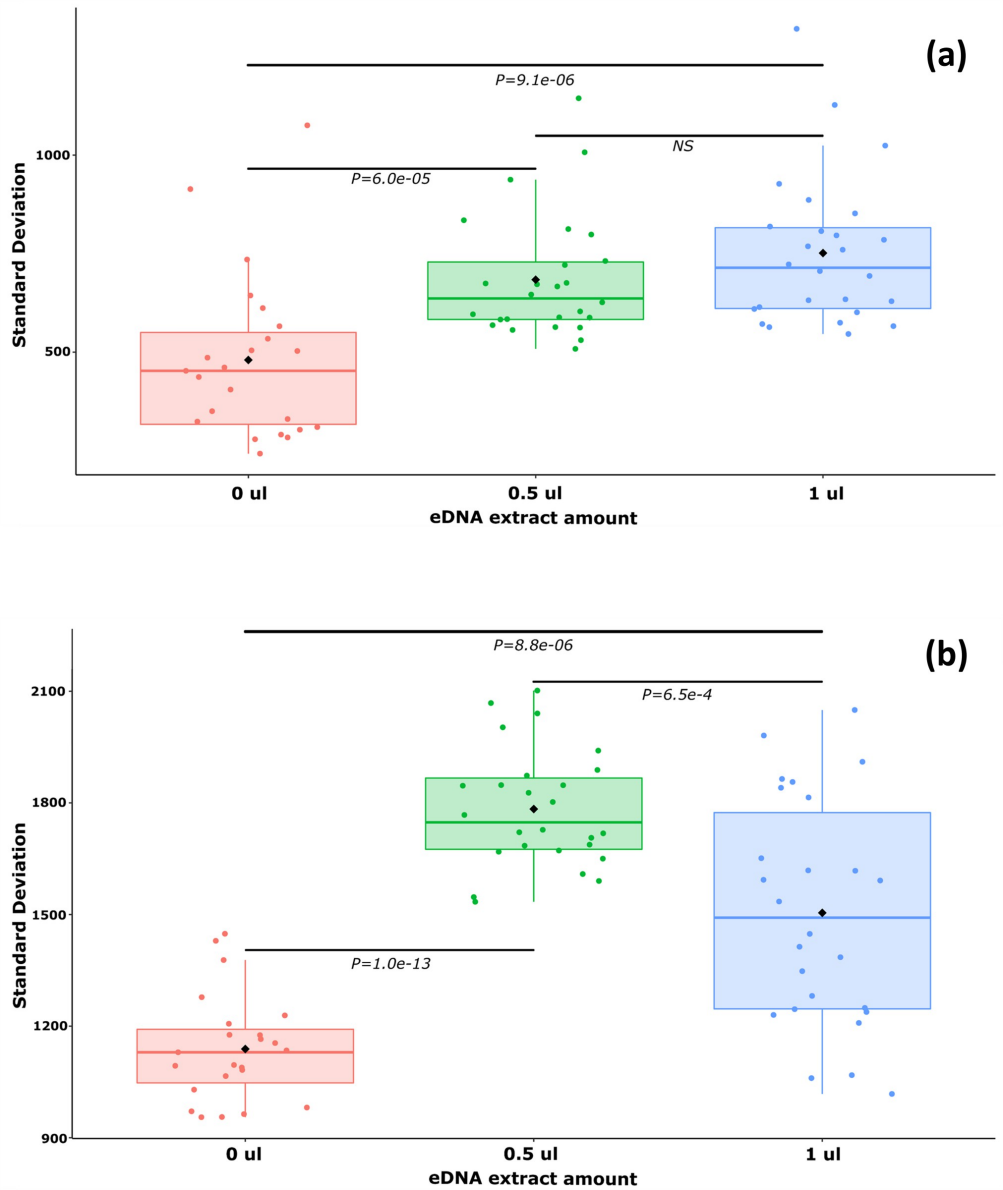
Boxplot representation of the standard deviations measured in (a) negative and (b) positive clouds for different amounts of eDNA samples (0, 0.5 and 1 μl) spiked in positive controls (*P*. *leniusculus* genomic DNA). Pairwise Wilcoxon test P values with Holm correction are indicated on horizontal bars (NS = non-significant).

The results of the experiment showed a clear inhibition effect impacting both (1) the difference of fluorescence amplitude between the positive and negative droplets clouds with a significant decrease in positive droplets fluorescence (Fig 3) and (2) the variability of the fluorescence amplitude in droplets from positive and negative clouds (Fig 4A and 4B).

### ddPCR *P*. *leniusculus* detection and DNA quantification in eDNA samples

The absolute concentrations of the target DNA for each replicate (n = 340) from the 34 targeted sites (Fig 1) were measured. The mean number of droplets generated was 18397, and all amplification products were rePCRed and sequenced. The resulting sequences were found to be specific i.e. matching *P*. *leniusculus* haplotypes from Genbank (ranging from 95.8% to 100% identity). None of the negative controls, neither field, extraction or amplification controls, yielded amplification.

However, 10 out of 78 no template controls produced artefactual amplifications ranging up to 3 droplets that could be accounted as positive. The LoB derived from these results was found at 2.25 cp/ul(copies per microliter) and the corresponding LoD at 3.89 cp/ul. Previous studies, already shown that ddPCR could generate background noise ranging from one to three positive droplets in no template controls [37–39]. Established from the dilution series of genomic DNA, the LoQ was reached at 5.98 cp/μl.

**Fig 5:**
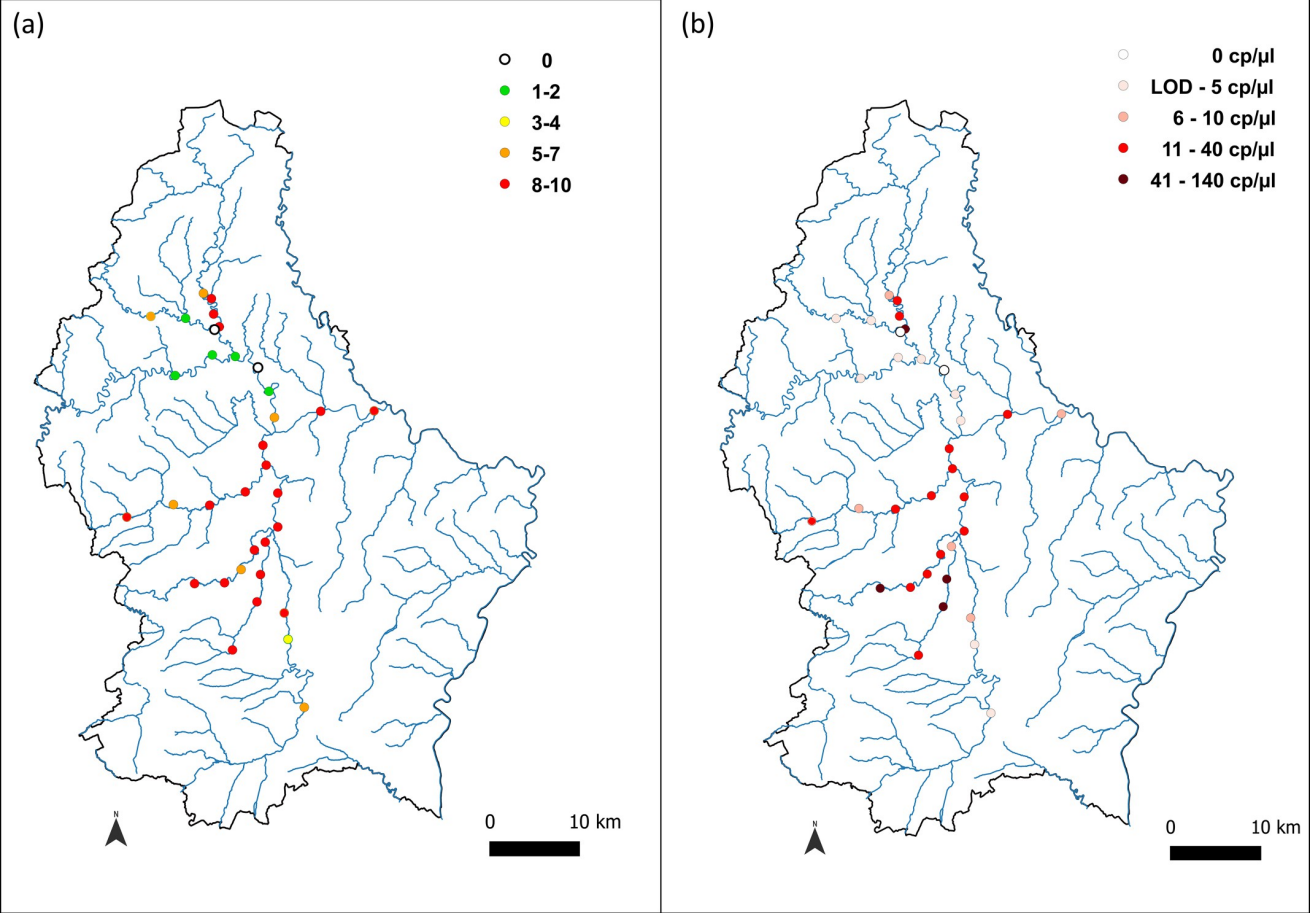
Map of the ddPCR results. (a) Number of successful amplification out of 10 replicates; (b) Mean concentrations per site in cp/μl.

The measured concentrations ranged from 6.01 to 163.77 cp/μl (S1 Table, Fig 5A). Out of 340 replicates, 243 were found positive for *P*. *leniusculus* detection (Fig 5A and 5B). At the site level, all but two (Su3 and Wi2) were found positive for the presence of *P*. *leniusculus* (Fig 5). No significant difference was found in target concentration between replicates sampled at 30 cm and 1.50m from the bank (Pairwise Wilcoxon test with Holm correction, P = 0.94).

‘Stars’ (extremely high fluorescence artefactual droplets) were found in 97 out of 340 replicates. The inclusion of these droplets for the positive droplets count would have caused 19 false positives and an overestimation bias in the DNA concentration measures in the samples from 1.19 to 10.9 cp/μl (Mean 1.94 cp/μl).

### Comparison with Quantasoft results

Compared to the method that accounted for the inhibition effect, the Quantasoft data treatment yielded 65 false negatives and 28 false positives at the replicate level (Table 1). Moreover it generated two false negatives at the site level for Sure and Wiltz rivers (Table 1). The global distribution of the measurements with the two methodologies were significantly different (Asymptotic two-sample Kolmogorov-Smirnov test, D = 0.30294, p-value = 5.618e-14). The concentrations measured with Quantasoft were significantly higher, either for replicates (Pairwise Wilcoxon test, p = 0.00029; range double threshold method 0–163.77 cp/ul, range Quantasoft 0–198 cp/ul) or sites mean concentrations (Pairwise Wilcoxon test, p = 0.016; range double threshold method 0–139 cp/ul, range Quantasoft 0.95–143.44 cp/ul).

**Table 1:** Inhibition impact in different river samples (reference value of the positive control = 6831.77), correlation between the DNA replicates’ concentrations produced with the two classifications methods (Quantasoft and the two threshold method developed here) and the number of false negatives and positives generated with Quantasoft at the replicate and site levels (the number in bracket is the value normalized to 40 replicates for rivers sampled in 7 sites instead of 4).

			Replicates			Sites
	Amplitude upper-lower threshold (fluorescence units)	Correlation of replicates’ concentrations	False neg.	False pos.	False neg.	
Alzette	7052.17	(R = 0.35; p = 0.0033)	23 (13)	1(0.6)		
Attert	12589.29	ns (R = 0.21; p = 0.2)	8			
Clerve	9604.90	ns (R = 0.14; p = 0.42)	3	1		
Eisch	7581.19	(R = 0.44; p = 0.0046)	5			
Mamer	8173.75	(R = 0.88; p = 4E-14)	3			
Sure	11460.80	ns (R = 0.09; p = 0.46)	20 (11)	15 (9)	1	
Wiltz	10085.05	ns (R = 0.27; p = 0.087)	3	11	1	

At the river level, no correlation was found in replicates’ concentration for Attert, Clerve, Sure and Wiltz rivers. These are the rivers in which the samples were most heavily affected by inhibition (Table 1). Samples less affected by inhibition (i.e. closer to positive control values), in Alzette, Eisch and Mamer, exhibited a significant linear correlation (Table 1) thus a certain level of linear proportionality in replicates’ concentrations between the two methods (to avoid an artificially increase of correlation statistics, DNA concentrations of 100 copies/μl and above were excluded from this analysis in Attert (n = 2) and Clerve (n = 5)).

## Discussion

### A threshold-based countermeasure against inhibition

ddPCR is reputed to be less sensitive to inhibition than other techniques applied to eDNA-based detection such as qPCR [19]. However, it was already found to be affected by inhibitors, which presence producedthe decrease of fluorescence in positive droplets [19, 20]. With a significant inhibition effect on the species-specific ddPCR assay targeted at *P*. *leniusculus* impacting both the position and the dispersion of the positive droplets cloud on the fluorescence scale, the data produced here further confirmed these findings. This could result in failure to discriminate positive droplets by applying a unique threshold determined either manually or through a software [29].

As a countermeasure, we proposed a distribution-based approach taking into account the inhibition effects by defining river-specific thresholds (sorting out the ‘rain’ i.e. droplets with intermediate fluorescence values between positive and negative droplets clouds). The method relies on the fact that the distribution of fluorescence droplet clouds follows a binomial distribution which can be approximate as normal [40]. Taking this inhibition effect into consideration is particularly important in eDNA ddPCR assays as DNA concentrations and thus the yield of positive amplification events are low. In this context, the consistent delineation of positive droplets clouds to the exclusion of ‘rain’ droplets is critical, as it could cause false positive and overestimate of the target’s DNA concentration.

As different levels of inhibition impacts can be expected from one river to another (see Table 1), data produced from each river have to be treated specifically concerning the two affected factors: (1) the amplitude of fluorescence between positive and negative clouds with the quenching of fluorescence in positive droplets and (2) the modification in dispersion of fluorescence values around the mean in both clouds. Therefore, concerning the delineation of positive droplets, it is crucial to consider the river specific inhibition effects which can be assessed through the river-specific correction factor proposed here (LTCF, Fig 2).

### Getting rid of the stars

In addition, the application of an upper threshold, corrected with the river-specific correction factors (UTCF, Fig 2) allowed to remove from the count of positive droplets the stars i.e. the extremely high fluorescence artefacts originating from droplets coalescence or from the amplification of non-target material [39, 41]. Although these ‘stars’ represents but a small fraction of the events in high concentration samples, and can therefore be disregarded, they can bias concentration measurements in very low concentration samples such as eDNA ones and even lead to false positive when the results approach the limit of detection. Here, ‘stars’ would have affected more than 28% of the replicates sample (n = 97), leading to both false positives and errors in the concentration measurements. Moreover, the method developed here allowed to deal as well with the inherent ddPCR artefact of baseline fluorescence shifting [42].

### A double threshold classification method for eDNA-based detection

Failure to account for the significant decrease in fluorescence induced by inhibitors in environmental samples such as water samples may cause false negative results as actual positive droplets would be expected to exhibit a higher fluorescence and thus would end up classified as ‘rain’. On the other hand, ‘stars’ could lead to false positives. The use of a consistent positive control-based methodology, with both an upper and a lower threshold, can be decisive to the analysis of eDNA target in ddPCR as templates often exhibit very low concentrations. For this very reason (i.e. low concentration templates), these issues can easily be overlooked when using automatic distribution-based solution such as Quantasoft (Bio-Rad) or manual ones that apply a same threshold to a complete set of samples, resulting in inconsistent or misleading results.

### Comparison with Quantasoft results

Here we compared our results with Quantasoft’s droplets sorting method which does not take inhibition effects into account and include ‘stars’ in the count of positive droplets. On the whole range of samples, it yielded a significant overestimation of the DNA concentrations but also produced 19% of false negatives and 8% of false positives (Table 1). This was due to low concentrations of the target DNA, which yielded scarce amplifications, combined with inhibition effects that provoked a shift in positive droplets’ fluorescence which were therefore interpreted as ‘rain’ by the Quantasoft software.

A significant correlation between the relative proportions of DNA concentrations determined by the Quantasoft and double-threshold methods was found in the Alzette, Eisch, and Mamer rivers (Table 1). These rivers were characterized by a low inhibition impact which likely allowed, to some extent, the recovery of comparable patterns in term of relative proportion in concentrations (Table 1). However, in Attert, Clerve, Sure and Wiltz rivers, which exhibited a higher inhibition effects on fluorescence amplitude, no correlation pattern was found between the results yielded by Quantasoft and our method. This highlights the need for eDNA studies to consider both inhibition effects, that significantly modify the fluorescence amplitude and thus ‘rain’ droplets designation, and the ‘stars’ droplets which can alter the measurements of concentrations and even the detection capability in low concentrations samples.

### Occurrence of *P*. *leniusculus* in Luxembourgish streams

This study allowed to investigate the occurrence of the invasive crayfish *P*. *leniusculus* in two of the main streams of Luxembourg (Alzette and Sure) along with five of their tributaries (Eisch, Attert, Mamer, Wiltz, Clerve). In only two sites, *P*. *leniusculus* was not detected. This confirms the status of this species as one of the main invaders in lotic freshwater ecosystems in Luxembourg. Indeed, the species is widespread throughout Europe and Luxembourg has been one of the countries where a large population of this species was introduced: 40 000 juveniles were imported from the main reproduction lake Simontorp in Sweden (lake) between 1972 and 1978, [43].

## Conclusion

In this study, in conjunction with an eDNA-based survey of *P*. *leniusculus* in main streams of Luxembourg, we proposed a new data treatment that can be used as a countermeasure against the degradation of the ddPCR signal due to inhibition. It enabled, to consistently delineate positive droplets from ‘rain’ but also from overshooting artefact droplets, ‘stars’, for low template concentrations. The proposed method was used to correct the various inhibition amounts in different rivers as well as the baseline fluorescence shifting. The systematic application of such countermeasures could contribute to homogenize the way ddPCR data are handled in eDNA-based detection and help avoiding both false positive and negative results due to inhibitors, extremely high fluorescence artefactual droplets and fluorescence baseline shifting. This is of particular importance in invasive species detection or endangered species monitoring as eDNA-based detection results can trigger costly and time-consuming management actions.

## Supporting information

S1 Table Concentration measured and volume filtered for each replicate. 0* = replicates in which 3 or less positive amplification events were observed; N^LOQ^ = Concentration measured under LOQ, replicates only retained for absence/presence information.
(XLSX)

S2 Table Upper and a Lower Threshold Correction Factor for the different target rivers. (XLSX)

S1 File Raw data. (ZIP)
